# Peroxiredoxin 6 overexpression attenuates lipopolysaccharide-induced acute kidney injury

**DOI:** 10.18632/oncotarget.17002

**Published:** 2017-04-10

**Authors:** Dong Hun Lee, Ju Ho Park, Sang Bae Han, Do young Yoon, Yu Yeon Jung, Jin Tae Hong

**Affiliations:** ^1^ College of Pharmacy and Medical Research Center, Chungbuk National University, Osong-eup, Heungduk-gu, Cheongju, Chungbuk, 361-951, Republic of Korea; ^2^ Department of Bioscience and Biotechnology, Bio/Molecular Informatics Center, Konkuk University, Hwayang-dong, Gwangjin-gu, Seoul 143-701, Republic of Korea; ^3^ Department of Pediatrics, School of Medicine, Emory University, Atlanta, GA 30329, USA; ^4^ Department of Dental Hygiene, Gwangyang Health Sciences University, Gwnagyang, Jeonnam 57764, Republic of Korea

**Keywords:** peroxiredoxin 6, acute kidney injury, reactive oxygen species, MAP kinase

## Abstract

Peroxiredoxin 6 (PRDX6) is a member of the PRDX family of antioxidant enzymes and correlated with inflammatory response. Therefore, we investigated the role of PRDX6 during lipopolysaccharide (LPS)-induced acute kidney injury. Both 3 months aged PRDX6-overexpressing transgenic mice (PRDX6 mice) and wild type (WT) mice had acute renal injury induced by intraperitoneal injection of LPS (10 mg/kg)., PRDX6 mice showed decreased mortality and renal injury following LPS challenge compared to WT mice. Furthermore, infiltration of macrophages, T-cells and neutrophils, and the number of apoptotic cells were more decreased by LPS treatment in PRDX6 mice than in WT mice. Because LPS induces reactive oxygen species (ROS) production which induces inflammation through c-Jun N-terminal Kinase (JNK) and p38 MAPK activation, we investigated ROS concentration and MAPK signaling pathway in the kidney of PRDX6 mice. As expected, LPS-induced oxidative stress was attenuated, and p38 MAPK and JNK activation was decreased in the kidney of PRDX6 mice. Inhibitory effect of PRDX6 on LPS-induced apoptosis and MAPK activation in the primary renal proximal tubular cells were overcome by treatment with PRDX6 inhibitor or hydrogen peroxide. These results suggest that PRDX6 overexpression inactivates p38 MAPK and JNK pathway through decrease LPS-induced ROS concentration in the kidney, resulting in inhibition of renal apoptosis and leukocyte infiltration and led to attenuation of LPS-induced acute kidney injury.

## INTRODUCTION

Acute kidney injury (AKI), so called acute renal failure (ARF) is a disease which resulting in rapid loss of function in kidney with severe tubular damage. It is a frequent and serious complication of sepsis and occurs approximately 19 percent of patient with moderate sepsis and 23 percent of patient with severe sepsis [1]. Acute kidney injury is often diagnosed on the basis of blood tests, elevated blood urea nitrogen level and creatinine level in kidney, or inability of kidney that produce sufficient amounts of urine [2].

Lipopolysaccharide (LPS) is a component of outer membrane in Gram-negative bacteria and is involved in pathogenesis of sepsis-induced acute kidney injury [3, 4]. LPS induces oxidative stress with increased reactive oxygen species (ROS) via activation of NADPH oxidase [5]. ROS are chemically reactive molecules containing oxygen; examples are peroxides, superoxide, hydroxyl radical, and singlet oxygen. ROS serves as intracellular signaling molecules to control the activation of NF-κB [6] and intracellular signaling pathways such as mitogen-activated protein kinases (MAPKs) [7]. Numerous reports demonstrated that ROS involved in sepsis-induced acute kidney injury [8–10]. Moreover, several studies showed that antioxidants ameliorate sepsis-induced acute kidney injury [11, 12]. Thus, ROS may play a critical role in sepsis-induced acute kidney injury.

Peroxiredoxins (PRDXs) are antioxidant enzymes that control cytokine-induced peroxides level and mediate signal transduction in mammalian cells. ROS can be scavenged by peroxidase and PRDXs represent a superfamily of nonseleno peroxidase [13]. Yang et al. reported that PRDX2 deficient mice showed increased susceptibility of LPS-induced lethal shock and intravenous injection with adenoviral PRDX2 gene into PRDX2 deficient mice rescued mice from LPS-induced lethal shock [14]. Li et al. reported that PRDX3 deficient mice also showed more severely LPS-induced lung injury [15]. Unlike the other members of PRDXs family with two reactive cysteines, Prdx6 has a single redox-active cysteine residue. However, PRDX6 may play protective role in LPS-induced tissue injury because PRDX6 deficiency exacerbates LPS-induced acute lung injury in mice through increasing oxidative stress [16]. PRDX6 has glutathione (GSH) peroxidase activity and its catalytic mechanism about peroxidase is based on GSH instead of thioredoxin for physiologic reductant [17]. *In vitro* studies have indicated that PRDX6 can interact with the π isoform of GSH S-transferase (GTSH), resulting in reduction of oxidized PRDX6 by GSH and the regeneration of active enzyme [18–20]. Thus, prdx6 performs a role in antioxidant defense by scavenge of hydrogen peroxide. Studies using various cell lines showed that overexpression of Prdx6 increases resistance to experimental oxidative stress [21, 22].

Because of these important physiologic roles, Prdx6 is a key enzyme for both antioxidant defense and phospholipid homeostasis. Therefore, we developed a model of LPS-induced acute kidney injury in prdx6-overexpressed mice and investigated the role of prdx6 in endotoxemic renal injury.

## RESULTS

### PRDX6 mice were resistance to LPS-induced endotoxin shock and acute kidney injury

**Figure 1:**
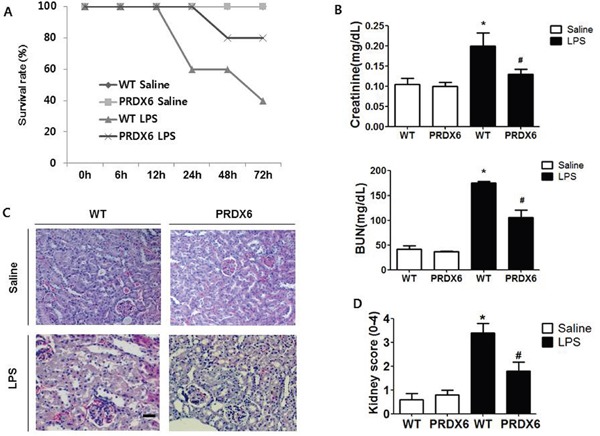
PRDX6 mice showed lower susceptibility to LPS-induced endotoxin shock and acute kidney injury **(A)** Survival rates of WT and PRDX6 mice after either LPS or saline administration. WT and PRDX6 mice were intraperitoneally injected with LPS (10 mg/kg) or saline. Survival rates were monitored for 72 hours. (n=10; *P* = 0.01 at 72 hours) **(B)** Renal function measured by blood urea nitrogen (BUN) and serum creatinine levels at 24 h after LPS and saline injection. ± SEM, *P < 0.05. (n = 5), saline versus LPS injected WT mice, ^#^*P* < 0.05. (n = 5), LPS injected WT versus PRDX6 mice. **(C)** Effect of PRDX6 on renal morphology in the acute kidney injury caused by LPS (Scale bars, 50μm). **(D)** Kidney scores that were obtained by blindly analyzing H&E stained kidney sections with at least 15 glomeruli of in each group. Glomerular hypercellularity was graded on a 0-4 scale in which 0 = normal glomeruli, 1 = <20%, 2 = 20-40%, 3 = 40-80% and 4= >80% abnormal glomeruli. ± SEM, *P < 0.05. (n = 5), saline versus LPS injected WT mice, ^#^*P* < 0.05. (n = 5), LPS injected WT versus PRDX6 mice.

To examine whether PRDX6 overexpression leads to resistance to endotoxin shock, we analyzed experimental endotoxin shock, induced by i.p. injection of LPS (10 mg/kg) in PRDX6-overexpressing transgenic mice (PRDX6 mice) and wild type (WT) mice at about 3 months of age. We showed that PRDX6 expression was increased in transgenic mice expectedly, and endogenous PRDX6 expression was not changed by LPS injection (Supplementary Figure 1). Forty percent of WT mice survived 3 day after LPS challenge, whereas eighty percent of PRDX6 mice survived (Figure 1A). Because PRDX6 mice showed protection to LPS-induced endotoxin shock, we examined LPS-induced renal injury in WT and PRDX6 mice. Blood urea nitrogen (BUN) and serum creatinine levels (markers of renal injury) were increased by LPS challenge and LPS-induced these levels were decreased in PRDX6 mice (Figure 1B). Histopathology studies revealed that PRDX6 mice showed decreased tubular damage and glomerular destruction in the kidney by LPS administration compared to WT mice (Figure 1C and 1D).

### PRDX6 overexpression attenuated LPS-induced renal apoptosis

**Figure 2:**
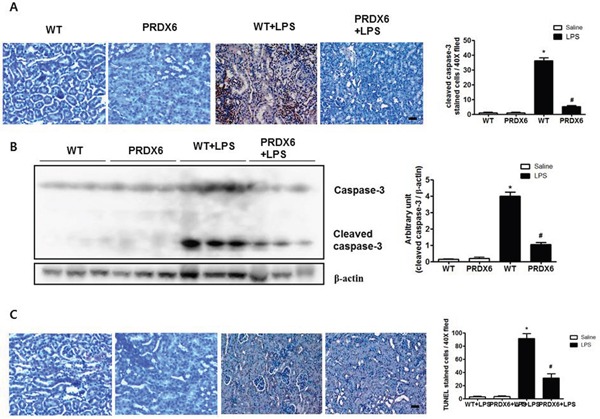
PRDX6 mice showed decreased apoptotic cell death in the kidney after LPS administration **(A)** Immunohistochemistry of cleaved caspase-3 and the number of reactive cells in the kidney section of WT and PRDX6 mice after LPS injection (Scale bars, 50μm). Cleaved caspase-3 stained cells in high power fields at 40X magnification per kidney of 5 different mice in each group were counted by light microscopy in a blinded fashion. ± SEM, *P < 0.05. (n = 5), saline versus LPS injected WT mice, ^#^*P* < 0.05. (n = 5), LPS injected WT versus PRDX6 mice. **(B)** Caspase-3 activity was determined in the total protein extracts of LPS-injected WT and PRDX6 mice kidney tissues by Western blotting. ± SEM, *P < 0.05. (n = 5), saline versus LPS injected WT mice, ^#^*P* < 0.05. (n = 5), LPS injected WT versus PRDX6 mice. **(C)** Immunohistochemistry of terminal deoxynucleotidyl transferase-mediated dUTP nick-end labeling (TUNEL) and the number of stained cells in the kidney section of WT and PRDX6 mice after LPS injection (Scale bars, 50μm). ± SEM, *P < 0.05. (n = 5), saline versus LPS injected WT mice, ^#^*P* < 0.05. (n = 5), LPS injected WT versus PRDX6 mice.

Because acute renal injury induced by LPS challenge was found to be more highly decreased in PRDX6 than in WT mice, we examined renal apoptosis as a potential contributing factor. Compared with the kidney tissue of WT mice, that of PRDX6 mice displayed a significantly decreased number of cleaved caspase-3 reactive cells (Figure 2A), and the decrease in caspase 3 cleavage in PRDX6 mice was confirmed by Western blot analysis (Figure 2B). The number of apoptotic cells was also smaller in the kidney of PRDX6 mice compared with the kidney of WT mice (Figure 2C).

### PRDX6 overexpression inhibited LPS-induced macrophage, T cells and neutrophils infiltration as well as JNK and p38 MAP kinase activation

**Figure 3:**
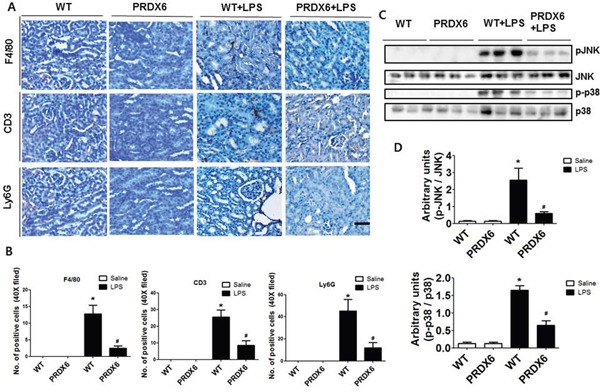
PRDX6 mice showed lower LPS-induced immune cells infiltration and activation of MAPK in the kidney than WT mice **(A)** Immunohistochemistry of infiltrated immune cells such as macrophages (F4/80), T cells (CD3) and neutrophils (Ly6G) in the kidney of LPS-injected WT and PRDX6 mice (Scale bars, 50μm). **(B)** Infiltrated macrophages (F4/80), T cells (CD3) and neutrophils (Ly6G) in the kidney of WT and PRDX6 mice. ± SEM, *P < 0.05. (n = 5), saline versus LPS injected WT mice, ^#^*P* < 0.05. (n = 5), LPS injected WT versus PRDX6 mice. **(C)** Activation of JNK and p38 MAP kinase was determined in the total protein extracts of LPS-injected WT and PRDX6 mice kidney tissues by Western blotting and **(D)** quantified blot data. ± SEM, *P < 0.05. (n = 5), saline versus LPS injected WT mice, ^#^*P* < 0.05. (n = 5), LPS injected WT versus PRDX6 mice.

Since immune cell infiltration is a major contributor to acute renal injury, we investigated the infiltration of leukocytes in the kidney of WT and PRDX6 mice 24 h after LPS challenge. We stained kidney sections for F4/80, a marker specific for macrophages; Ly6G, a marker specific for neutrophils; and CD3, a marker specific for T cells. The numbers of infiltrated macrophages, T cells and neutrophils were decreased in the kidney of LPS-injected PRDX6 mice compared with that in the kidney of LPS-injected WT mice (Figure 3A and 3B). Because LPS induces MAP kinase activation and these signaling pathways promote inflammatory response and apoptosis, we measured the activation of MAP kinase in the kidney. Compared with the kidney of WT mice, that of PRDX6 mice showed significantly lower amounts of activated p38 MAP kinase and JNK, but not of ERK, following LPS challenge (Figure 3C and 3D).

### LPS-induced oxidative stress decreases in PRDX6 mice

**Figure 4:**
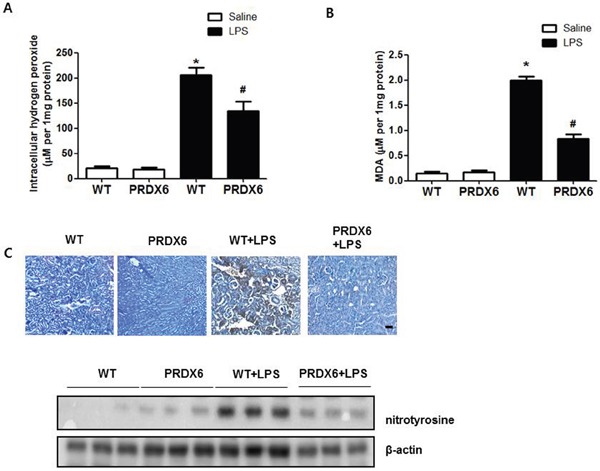
PRDX6 mice showed lower LPS-induced oxidative stress in the kidney than WT mice **(A)** Intracellular hydrogen peroxide levels in the kidney of WT mice and PRDX6 mice injected with LPS (10 mg/kg) at 24 h. ± SEM, *P < 0.05. (n = 5), saline versus LPS injected WT mice, ^#^*P* < 0.05. (n = 5), LPS injected WT versus PRDX6 mice. **(B)** MDA levels in the kidney of WT mice and PRDX6 mice injected with LPS (10 mg/kg) at 24 h. ± SEM, *P < 0.05. (n = 5), saline versus LPS injected WT mice, ^#^*P* < 0.05. (n = 5), LPS injected WT versus PRDX6 mice. **(C)** Immunohistochemistry analysis and Western blot analysis of nitrotyrosine-positive protein in the kidney of WT mice and PRDX6 mice injected with LPS (10 mg/kg) at 24 h.

Because reactive oxygen species (ROS) play an important role in sepsis-induced acute kidney injury through activates JNK and p38 MAPK resulting in inflammation and apoptosis [8–10, 23], we investigated the effect of PRDX6 on oxidative stress in LPS-induced kidney tissues of mice. We observed that hydrogen peroxide levels were lower in the kidney of LPS-injected PRDX6 mice compared to LPS-injected WT mice (Figure 4A). Malondialdehyde (MDA) is a naturally occurring product of lipid peroxidation and is a marker for oxidative stress. The levels of MDA in the kidney were also lower in the liver of LPS-injected PRDX6 mice compared to WT mice (Figure 4B). Moreover, nitrotyrosine is also marker for oxidative stress because it is formed in the presence of the active metabolite NO and an oxidative stress. We observed that the number of nitrotyrosine positive cells and expression of nitrotyrosine were also decreased in the kidney of LPS-injected PRDX6 mice compared to LPS-injected WT mice (Figure 4C).

### PRDX6 decreased LPS-induced apoptosis in primary renal proximal tubular cells via ROS removal

**Figure 5:**
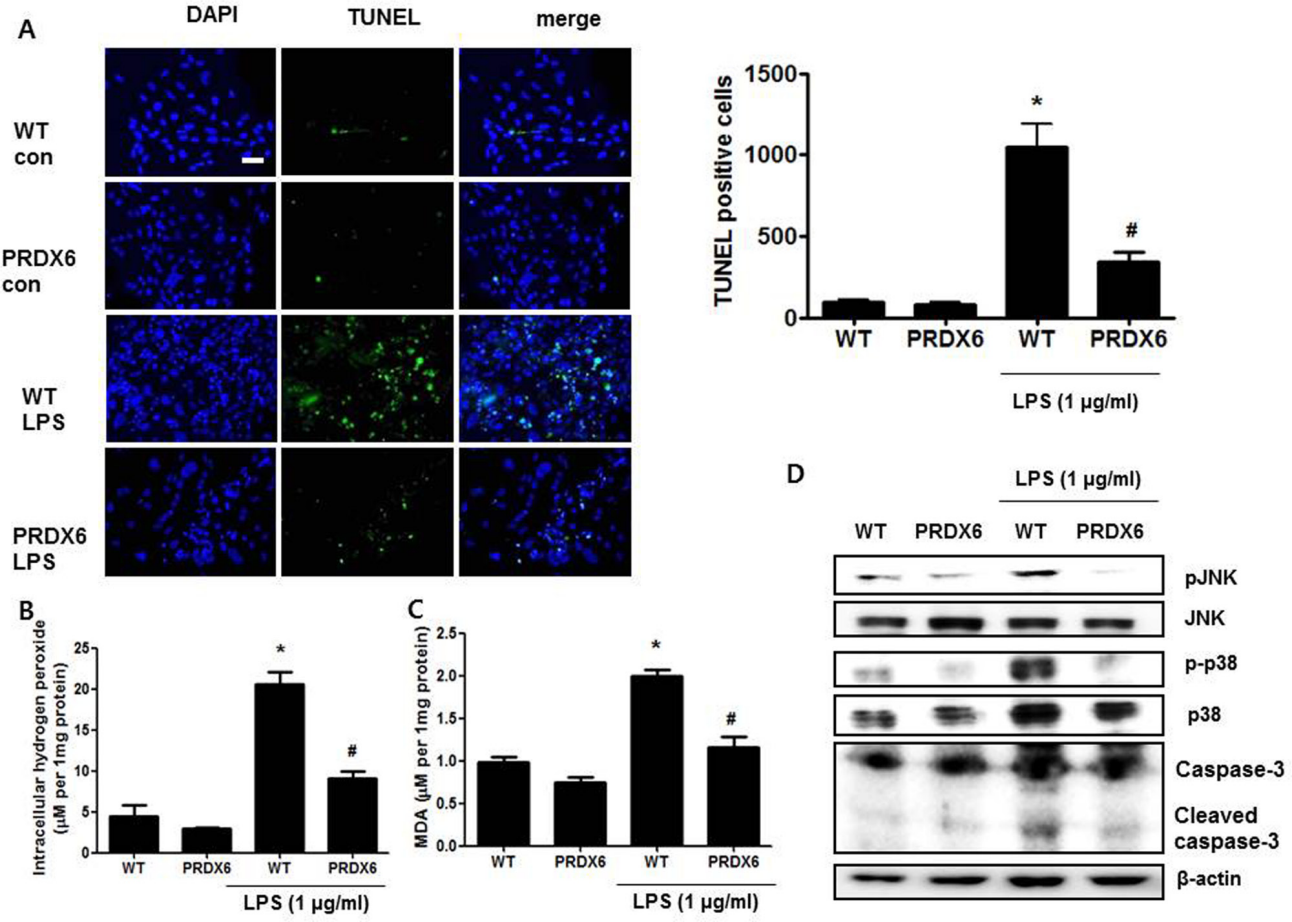
LPS-induced apoptotic cells and activation of MAPK were decreased in the primary renal proximal tubular cells from PRDX6 mice compared to cells form WT mice **(A)** Cells were treated with 1 μg/ml LPS. After 24 h, cells were fixed by formalin and then preformed TUNEL staining (Scale bars, 20μm), and quantified TUNEL positive cells (per 20,000 DAPI stained cells). ± SEM, *P < 0.05, control versus LPS treated cells from WT mice (n = 5), ^#^*P* < 0.05, LPS treated cells from WT mice versus LPS treated cells from PRDX6 mice (n = 5). **(B)** Intracellular hydrogen peroxide levels and **(C)** MDA levels in the primary renal proximal tubular cells from WT and PRDX6 mice treated with LPS (1 μg/ml) at 24 h. ± SEM, *P < 0.05, control versus LPS treated cells from WT mice (n = 5), ^#^*P* < 0.05, LPS treated cells from WT mice versus LPS treated cells from PRDX6 mice (n = 5). **(D)** Activation of JNK and p38 MAP kinase was determined in LPS-treated primary renal proximal tubular cells from WT and PRDX6 mice by Western blotting.

**Figure 6:**
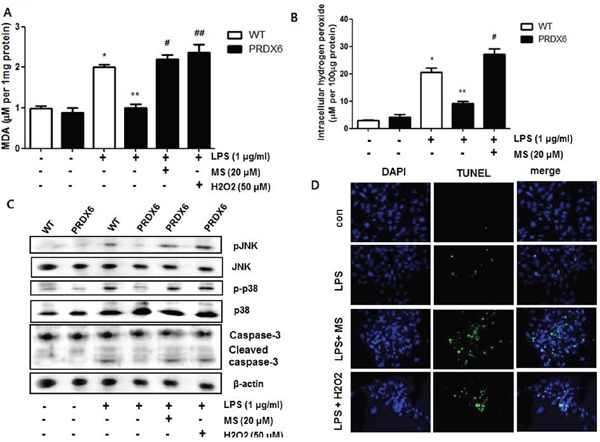
Effects of PRDX6 inhibitor or hydrogen peroxide on LPS-induced oxidative stress and apoptotic cell death of the primary renal proximal tubular cells from PRDX6 mice Cells were treated with 1 μg/ml LPS in the presence or absence PRDX6 inhibitor, MS (20 μM) or hydrogen peroxide (50 μM). **(A)** MDA level was measured in primary renal proximal tubular cells from WT and PRDX6 mice 24h after LPS addition. *P < 0.05, control versus LPS treated cells from WT mice (n = 5),^**^P < 0.05, LPS treated cells from WT mice versus LPS treated cells from PRDX6 mice (n = 5), ^#^*P* < 0.05, pre-treated with versus without MS and LPS treated cells from PRDX6 mice (n = 5), ^##^*P* < 0.05, pre-treated with versus without hydrogen peroxide and LPS treated cells from PRDX6 mice (n = 5). **(B)** Intracellular hydrogen peroxide level was measured in primary renal proximal tubular cells from WT and PRDX6 mice 24h after LPS addition *P < 0.05, control versus LPS treated cells from WT mice (n = 5),^**^P < 0.05, LPS treated cells from WT mice versus LPS treated cells from PRDX6 mice (n = 5), ^#^*P* < 0.05, pre-treated with versus without MS and LPS treated cells from PRDX6 mice (n = 5). **(C)** Effect of PRDX6 inhibitor, MS (20 μM) or hydrogen peroxide (50 μM) on LPS-induced JNK and p38 MAPK activation in the primary renal proximal tubular cells from PRDX6 mice. **(D)** Effect of PRDX6 inhibitor, MS (20 μM) or hydrogen peroxide (50 μM) on LPS-induced apoptotic cell death in the primary renal proximal tubular cells from PRDX6 mice determined by TUNEL staining (Scale bars, 20μm).

Because we observed that PRDX6 mice showed decreases in renal injury and oxidative stress in the kidney tissues after LPS challenge compared to WT mice, we investigated that the effect of PRDX6 overexpression on renal apoptosis and oxidative stress in the primary renal proximal tubular cells from WT mice or PRDX6 mice. Like kidney tissue, Apoptotic cells were increased in the primary renal proximal tubular cells from WT mice at 24 h following LPS treatment; whereas, LPS-induced apoptotic cells were lower in the primary renal proximal tubular cells from PRDX6 mice compared to cells from WT mice (Figure 5A). Moreover, LPS-induced hydrogen peroxide levels, MDA levels, caspase-3 cleavage and activation of JNK and p38 MAPK were also decreased in the primary renal proximal tubular cells from PRDX6 mice compared to cells from WT mice (Figure 5B-5D). Furthermore, PRDX6 knock down condition using PRDX6 si-RNA transfection in primary renal proximal tubular cells increased LPS-induced ROS production and MAPK activation (Supplementary Figure 2). Because PRDX6 have nonseleno-glutathione peroxidase function and can degrade hydrogen peroxide which increase lipid peroxidation and MAPK activation results in renal injury, primary renal proximal tubular cells from PRDX6 mice were treated with LPS in either the presence or absence of mercaptosuccinate (MS) which inhibited peroxidase activity of PRDX6 [24]. LPS-induced hydrogen peroxide level and MDA level were significantly decreased in primary renal proximal tubular cells from PRDX6 mice compared to cells from WT mice; however, its decreased levels were restored by MS (20 μM) or hydrogen peroxide (50 μM) pre-treatment (Figure 6A and 6B). Next, we examined that MS or hydrogen peroxide restore oxidative stress-induced JNK and p38 MAPK activation in primary renal proximal tubular cells from PRDX6 mice. LPS-induced JNK and p38 MAPK activation were significantly decreased in primary renal proximal tubular cells from PRDX6 mice compared to cells from WT mice; however, its inhibited effects were restored by MS (20 μM) or hydrogen peroxide (50 μM) pre-treatment (Figure 6C). Furthermore, caspase-3 cleavage and apoptotic cells were also increased in the primary renal proximal tubular cells from PRDX6 mice at 24 h following LPS treatment by MS (20 μM) or hydrogen peroxide (50 μM) pre-treatment (Figure 6C and 6D). And, NF-kB pathway play a key role in the LPS induced inflammatory response, as well as apoptosis, We showed that LPS-induced the DNA binding activity of NF-κB was decreased in primary renal proximal tubular cells from PRDX6 mice compared to cells from WT mice; however, its inhibited effects were restored by MS (20 μM) or hydrogen peroxide (50 μM) (Supplementary Figure 3).

## DISCUSSION

Oxidative stress is a redox imbalance between production and removal of oxidant and causative factor of various diseases including sepsis [25, 26]. ROS, which are oxidative mediated molecules, are reactive molecules and free radicals derived from molecular oxygen. Hydrogen peroxide is one of important ROS and many studies reported that LPS increases hydrogen peroxide production. PRDXs are known as antioxidant enzyme and several studies reported that play a protective role in LPS-induced tissue injury. However, the role of PRDX6 in acute renal injury caused by septic shock has not previously been studied. Therefore, we compared the characteristics of LPS-induced acute renal injury between WT mice and PRDX6 mice. The main finding of our study is that PRDX6 mice showed decreased lethality and acute renal injury following LPS-induced septic shock.

Oxidative stress is closely associated with acute kidney injury, and it is increased in patient with acute renal failure [27]. Several studies showed that ROS play an important role in LPS-induced acute kidney injury [10, 23]. Xu et al. reported that MDA level, a marker of lipid peroxidation, was elevated and nitrotyrosine, a marker of protein nitration, was detected in the kidney of LPS-treated mice, and treatment of Vitamin D3 which is a classical antioxidant ameliorated LPS-induced acute kidney injury [12]. In several studies, PRDXs is associated with endotoxin-induced inflammation and septic shock. Bast et al. reported that LPS induces PRDX1 gene expression via JNK signaling pathway, and increased PRDX1 may protect against oxidative stress-related injury [28]. PRDX2 deficiency as well as PRDX3 deficiency were increased LPS-induced septic shock in mice [14, 15], and circulating PRDX4 level was reported as oxidative stress marker in patient with sepsis [29]. Like other PRDXs members, PRDX6 can remove hydrogen peroxide because it have peroxidase activity [17], and Yang et al. reported that PRDX6 deficient mice show that more increased LPS-induced MDA and hydrogen peroxide levels in the lung compared to WT mice [16]. In our data, PRDX6 mice showed that decreased LPS-induced MDA level and protein nitration in the kidney. Moreover, LPS-induced hydrogen peroxide level was also decreased in PRDX6 mice compared to WT mice. These results suggest that PRDX6 decreased LPS-induced oxidative stress in the kidney through removal of hydrogen peroxide.

MAP kinases are involved in the renal inflammation and injury. JNK signaling is mediated by various insults including ischemia/reperfusion, ureteric ligation, immune-mediated injury, and hyperglycaemia [30, 31]. JNK activation has been demonstrated in several nephritis models [32], and JNK inactivation suppressed renal inflammation, tubular apoptosis and interstitial fibrosis [33, 34]. Karnellis et al. reported that JNK inhibitor pre-administration prevented tubular damage and renal dysfunction in ischemia/reperfusion model [35]. Moreover, macrophage-mediated renal injury is dependent on JNK pathway [36]. p38 is another MAPK protein and p38 MAPK signaling activated by LPS and IL-1 [37]. In autoimmune renal disease model, inhibition of p38 MAPK reduced the severity of disease through reduced infiltration of leukocytes, diminished cytokines which are known to promote renal injury [38]. Several studies reported that LPS or cisplatin induced acute renal injury via p38 MAPK pathway and inhibition of p38 MAPK signaling reduced acute kidney injury [39, 40]. Moreover, we previously reported that C-C chemokine receptor 5 deficient mice showed higher susceptibility LPS-induced acute kidney injury through increased p38 MAPK activation [41]. JNK and p38 MAPK signaling is associated with leukocytes infiltration, cell survival, necrosis and apoptosis in the kidney [36, 42–44]. JNK activation by ROS in proximal tubular epithelial cells [45], and oxidative stress also activated p38 MAPK signaling in the kidney [46]. Ramesh et al. reported that dimethylthiourea, an oxygen radical scavenger, completely protected cisplatin-induced acute kidney injury by p38 MAPK inactivation [47]. In our data, LPS-injected PRDX6 mice showed that decreased JNK and p38 MAPK activation and leukocytes infiltration in the kidney compared to WT mice. Furthermore, LPS-injected PRDX6 mice showed that decreased apoptotic cells in the kidney compared to WT mice. These results suggest that PRDX6 overexpression attenuated leukocyte infiltration and renal injury through decreased MAPK activation by ROS scavenge.

Increased ROS level also induces apoptosis in renal cells through MAPK pathway [48, 49]. To confirm the effects of PRDX6 on LPS-induced renal apoptosis *in vitro*, we investigated LPS-induced apoptosis, oxidative stress and JNK and p38 MAPK activation in primary renal proximal tubular cells from WT and PRDX6 mice. We observed that LPS-induced apoptosis, oxidative stress and JNK and p38 MAPK activation were lowered in primary renal proximal tubular cells from PRDX6 mice compared to cells from WT mice. Decreased hydrogen peroxide level in LPS-treated primary renal proximal tubular cells from PRDX6 mice was restored by MS which inhibits peroxidase activity of PRDX6. Moreover, MS or hydrogen peroxide pre-treatment overcame decreased MDA level and apoptosis in LPS-treated primary renal proximal tubular cells form PRDX6 mice. Furthermore, NF-κB signaling is correlated with inflammation, apoptosis and ROS [50–52]. We showed that PRDX6 inactivate LPS-induced NF-κB and these inactivated effects were restored by MS or hydrogen peroxide. These results suggest that PRDX6 attenuates LPS-induced renal apoptosis through removal of hydrogen peroxide.

In summary, our results suggest that PRDX6 overexpression attenuates LPS-induced acute kidney injury. This effect may result from lower MAPK activation through decreased oxidative stress by removal hydrogen peroxide.

## MATERIALS AND METHODS

### Animals

The C57BL/6J-Tg (PRDX6) mice were purchased from the Jack-son Laboratory. The mice were housed and bred under specific pathogen free conditions at the Laboratory Animal Research Center of Chungbuk National University, Korea. The C57BL/6J wild type (WT) mice and PRDX6 mice, that were used, had matched ages (about 3 months old). To generate endotoxemic ARF model, Mice were intraperitoneally (i.p.) injected with 10 mg/kg LPS from *Escherichia coli* 0111:B4 (Sigma, St. Louis, MO), and then we monitored 10 mice of each group for survival rate analysis until 72hr after LPS injection as well as 5 mice of each group were sacrificed at 24 h after LPS injection for analysis of pathology. All studies were approved by and performed according to the ethical guidelines by the Chungbuk National University Animal Care Committee (CBNU-523-13-01).

### The serum chemistry measurements

Mice were anesthetized with an overdose of pentobarbital (100 mg/kg) and blood was taken by heart puncture. Serum levels of BUN and creatinine were measured at laboratory animal research center in Chungbuk National University.

### Histological techniques

For histological processing, kidney tissues were fixed in phosphate buffer containing 10% formaldehyde and decalcified with EDTA. Fixed tissues were processed by routine methods to paraffin blocks. Specimens were sectioned at 4 μm and stained with H&E.

### Cell culture

Primary renal proximal tubular cells cultures from murine kidney were established as described previously [53]. WT and PRDX6 mice at a time were sacrificed by halothane inhalation. The mice were doused with 70% ethanol to minimize contamination of the primary cultures. Kidneys were removed from both mice using scissors and forceps soaked in 70% ethanol, and as each organ was removed it was immediately placed in a 100-mm tissue culture dish containing 10 ml of sterile PBS. Kidneys were minced separately into 1-mm cubes using razor blades dipped in 70% ethanol. The minced tissues were transferred into sterile 15-ml conical tubes containing sterile PBS. After allowing the minced tissue pieces to settle, the PBS was aspirated, and the tissues washed once more with sterile PBS. Twenty milliliter of Dulbecco's modified Eagle (DMEM) medium containing collagenase (2.5 mg/ml, Sigma, St. Louis, MO) and DNase I (50ul, Invitrogen, Carlsbad, CA) was added, and the tissue was incubated with rocking at 37°C for 3 h. After the incubation, collagenase-digested tissue and dissociated cells from tissue were centrifuged at 800 g for 5 min then rejected the supernant. The resulting pellet was gently pressed through a 100 μm cell strainer (BD, Franklin Lakes, New Jersey). The filtered cells were washed by DMEM medium and plated into 100 mm^2^ dishes. For obtain tubular fraction [54], density-gradient centrifugation of the pellet was then performed by resuspension in 25 ml of DMEM with 45% (vol/vol) sterile Percoll solution in 50 ml centrifugation tubes and centrifugation at 5525 × g for 30 min at 4°C (without braking). After centrifugation, the tubule fractions were collected from the top layer of the Percoll solution. The tubule fraction was washed once in 20 ml ice-cold DMEM medium at 300 × g for 5 min at 4°C and resuspended for further experiments. Cells were treated with LPS from *Escherichia coli* 0111:B4 (Sigma, St. Louis, MO) (1 μg/ml) for the indicated time and then were harvested. To examine the role of PRDX6 on renal apoptosis and ROS removal, cells were pre-treated inhibitor of PRDX6 peroxidase activity (20 μM mercaptosuccinate, Sigma, St. Louis, MO) or hydrogen peroxide (50 μM, Sigma, St. Louis, MO) for 2 h and then treated LPS from *Escherichia coli* 0111:B4 (Sigma, St. Louis, MO) (1 μg/ml) for 24 h.

### Western blot analysis

Homogenized kidney tissues and primary renal proximal tubular cells lysed by protein extraction solution (PRO-PREP, iNtRONBiotechnology, Korea) containing protease inhibitor cocktail (Calbiochem, Germany) and phosphatase inhibitor cocktail (Roche, Germany). Total proteins (30 μg) were separated by SDS-PAGE and transferred to a PVDF membrane (Millipore, Billerica, MA). The membrane was blocked with 5% skim milk overnight and then incubated with primary antibodies (diluted 1:1000) for 1 h at room temperature. The membranes were immunoblotted with the following primary antibodies: mouse monoclonal antibodies directed against, β-actin (Santa Cruz Biotechnology, Dallas, Texas, USA) and Ly6G (Biolegend, San diego, CA, USA), and against caspase-3, JNK, phospho-JNK, p38, phosphor-p38 (Cell Signaling Technology, Beverly, MA, USA), primary rat monoclonal antibody directed against F4/80, nitrotyrosine (Santa Cruz Biotechnology, Dallas, Texas, USA) primary rat monoclonal antibody directed against nitrotyrosine, CD3 (Abcam, UK). After washing with Tris-buffered saline containing 0.05% Tween-20 (TBST), the membrane was incubated with horseradish peroxidase-conjugated secondary antibodies (diluted 1:3000) for 1 h at room temperature. Binding of antibodies to the PVDF membrane was detected with enhanced chemiluminescence solution (Amersham Bioscience, UK) and X-ray film (AGFA, Belgium).

### Immunohistochemistry

All specimens were fixed in formalin and embedded in paraffin for examination. Sections (4 μm thickness) were stained with H&E and analyzed by immunohistochemistry using primary mouse monoclonal antibodies directed against Ly-6G/Ly6c(Gr-1) (1: 100 dilution, Biolegend, San diego, CA, USA), against caspase-3 ( 1: 100 dilution, Cell Signaling Technology, Beverly, MA, USA), and against CD3 (Abcam, UK), primary rat monoclonal antibody directed against F4/80 (1: 100 dilution, Santa Cruz Biotechnology, Dallas, Texas, USA), and secondary horseradish peroxidase conjugated anti-mouse and anti-rat antibodies.

### Oxidative stress assay

To perform assay, the kidney tissue or primary renal proximal tubular cells were homogenized, then normalized to protein concentration. We measured hydrogen peroxide level using OxiSelect Hydrogen Peroxide Assay kit (Cell Biolabs, SD, CA). This kit measured hydrogen peroxide level using horseradish peroxide (HRP) and non-fluorescent ADHP (10-Acetyl-3,7-dihydroxyphenoxazine) which react with hydrogen peroxide to produce highly fluorescent Resorufin, and the Resorufin product can be read by a fluorescent microplate reader with excitation of 530-560 nm and emission of 590 nm. The MDA level was measured using TBARS assay kit (cayman chemical, USA). The thiobabituric acid (TBA) react with MDA to produce MDA-TBA adduct under high temperature (90-100 °C) and this acidic conditions is measured colorimetically at 530-540 nm.

### Terminal deoxynucleotidyl transferase-mediated dUTP nick-end labeling (TUNEL) staining

Apoptosis of kidney tissues and primary renal proximal tubular cells were identified by TUNEL staining with an *in situ* cell death detection kit (Roche, Germany).

### Gel electromobility shift assay (EMSA)

DNA binding activity of nuclear factor kappa B (NF-κB) was determined by gel mobility shift assay. Gel electromobility shift assay (EMSA) was performed according to the manufacturer's recommendation (Promega, Madison, WI). The cells were briefly homogenized in 200 μL of solution A (10 mM HEPES (pH 7.9), 1.5mMMgCl2, 10 mM KCl, 0.5mM dithiothreitol, and 0.2mM phenylmethylsulfonylfluoride), incubated on ice for 6 min, and then centrifuged at 6,000 rpm for 6 min. Pelleted nuclei were re-suspended in solution C (solution A supplemented with 420 mM NaCl and 20% glycerol) and incubated on ice with vigorous vortexing every 5min for 20min. The re-suspended pellet was centrifuged at 15,000 rpm for 15 min and the resulting nuclear extract supernatants were collected in a chilled micro tube. Consensus oligonucleotides were end-labeled using T4 polynucleotide kinase and [P^32^]-ATP for 10 min at 37°C. Gel shift reactions were assembled and incubated at room temperature. Subsequently, 1 μL of gel loading buffer was added to each reaction and loaded onto a 6% non-denaturating gel. The gel was subjected to electrophoresis until the dye was four-fifths of the way down the gel. The gel was dried for 2 h at 80°C and exposed to film overnight at −70°C.

### Statistical analysis

The experiments were conducted either in triplicate, and all experiments were repeated at least three times with similar results. The data were expressed as the means ± SEM. Differences in mean were analyzed by *t* test or ANOVA, and *P* value of <0.05 was considered significant.

## SUPPLEMENTARY MATERIALS FIGURES


