# Supplementary material for: Peroxiredoxin 6 overexpression attenuates lipopolysaccharide-induced acute kidney injury
Source: Oncotarget. 2017 Apr 10;8(31):51096–107. doi: 10.18632/oncotarget.17002 (PMC5584234; doi:10.18632/oncotarget.17002)
Supplement: Supplementary file 1 [file oncotarget-08-51096-s001.pdf]

Peroxiredoxin 6 overexpression attenuates lipopolysaccharide-induced acute kidney injury

SUPPLEMENTARY FIGURES

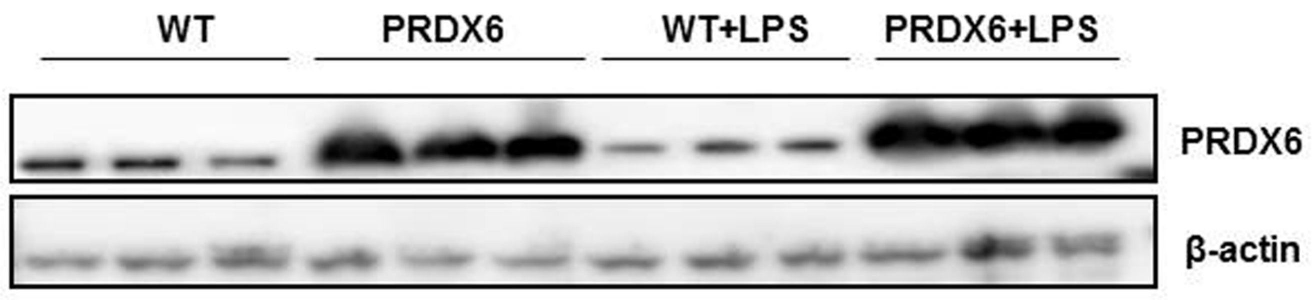

Supplementary Figure 1: The expression of PRDX6 of the kidney tissue in saline or LPS injected WT and PRDX6 mice.

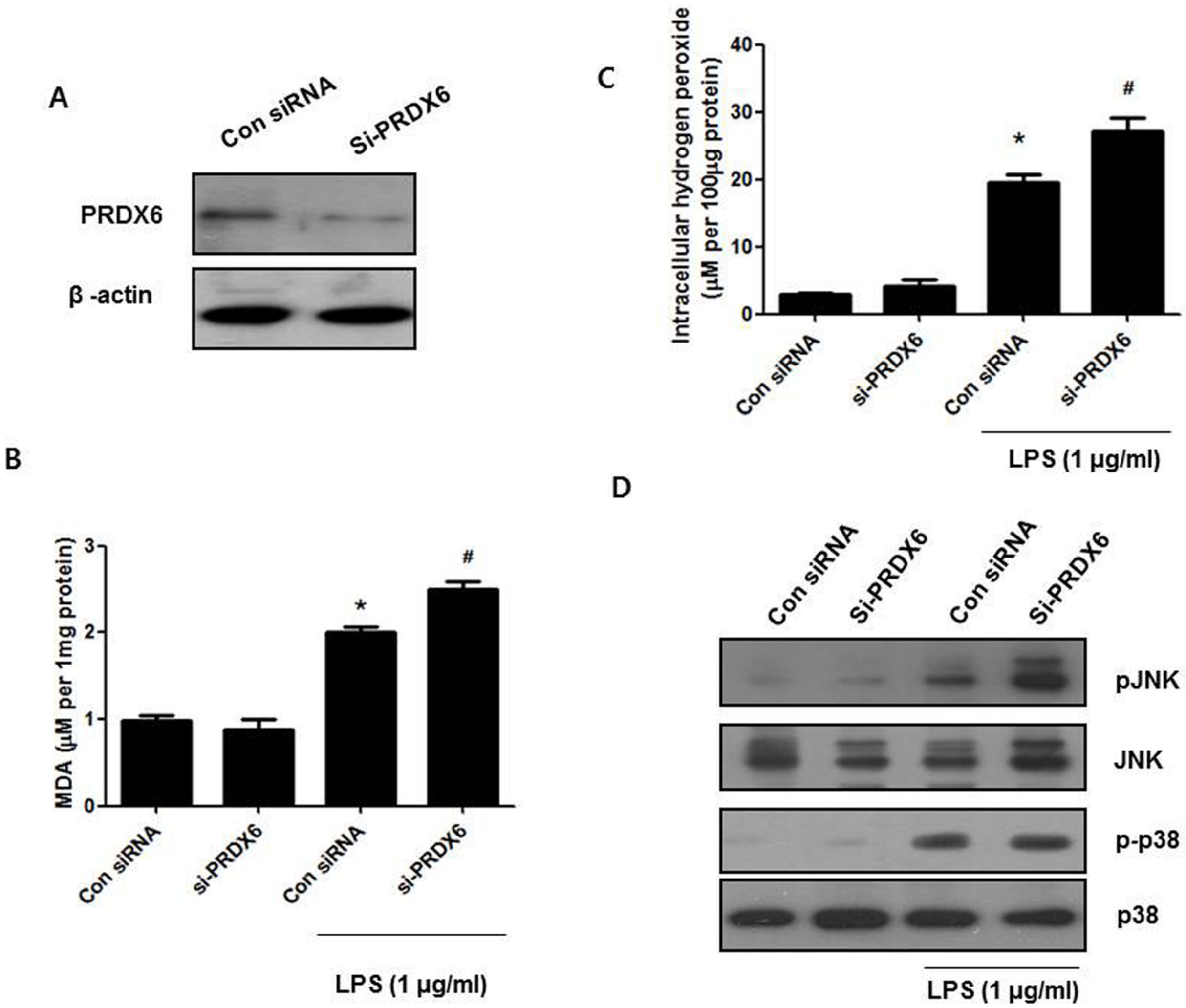

Supplementary Figure 2: Effects of PRDX6 knock down in LPS-treated primary renal proximal tubular cells. (A) The expression of PRDX6 in primary renal proximal tubular cells transfected with control or PRDX6 si-RNA **(B)** Intracellular hydrogen peroxide levels and **(C)** MDA levels in the primary renal proximal tubular cells transfected with control or PRDX6 si-RNA then treated with LPS (1 μg/ml) at 24 h. ± SEM, $^{*}P < 0.05$, control versus LPS treated cells from WT mice (n = 5), $^{\#}P < 0.05$, LPS treated cells from WT mice versus LPS treated cells from PRDX6 mice (n = 5). **(D)** Activation of JNK and p38 MAP kinase was determined in LPS-treated primary renal proximal tubular cells from WT and PRDX6 mice by Western blotting.

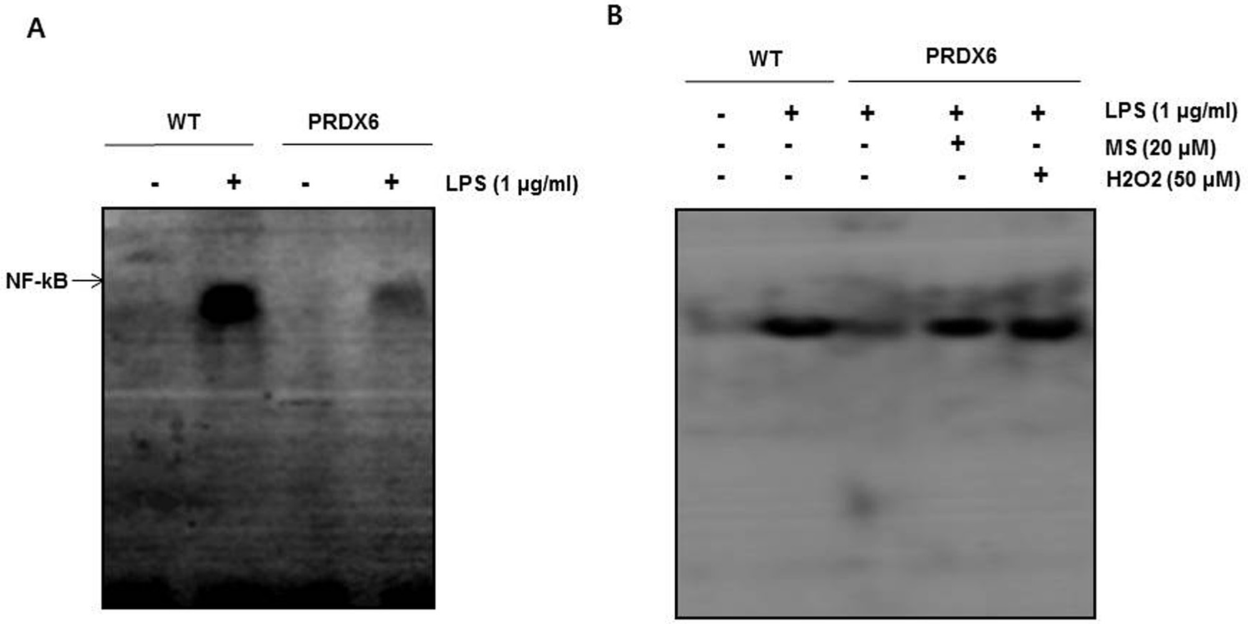

Supplementary Figure 3: Effects of PRDX6 on LPS-induced NF-κB signaling of the primary renal proximal tubular cells from PRDX6 mice. **(A)** Effects of PRDX6 on NF-κB DNA-binding activities measured by EMSA in LPS-treated primary renal proximal tubular cells from WT or PRDX6 mice. **(B)** Effect of PRDX6 inhibitor, MS (20 μM) or hydrogen peroxide (50 μM) on LPS-induced NF-κB DNA-binding activities in the primary renal proximal tubular cells from PRDX6 mice measured by EMSA.
